# Burnout across boundaries: Can parental burnout directly or indirectly influence work outcomes?

**DOI:** 10.1007/s12144-021-02687-3

**Published:** 2022-01-22

**Authors:** Thomas Rhys Evans, Isabelle Roskam, Florence Stinglhamber, Moïra Mikolajczak

**Affiliations:** 1 School of Human Sciences, University of Greenwich, London, UK; 2 Université Catholique de Louvain, Louvain, Belgium

**Keywords:** Parental burnout, Job burnout, Depression, Job satisfaction, Turnover intentions, Counterproductive work behavior

## Abstract

Burnout, while historically considered a work-related condition, can be associated with parenting where it can have direct impacts upon parental outcomes and one’s personal resources such as mental health. However, little is known about the domain-incongruent effects of burnout and thus whether parental burnout can manifest within the workplace. The current study uses longitudinal data collected from 499 parents over three intervals across an 8-month period to explore two possible mechanisms. Firstly, a direct relationship is explored by considering whether parental burnout provides incremental validity above job burnout in the prediction of three work outcomes: job satisfaction, turnover intentions, and counterproductive work behaviors. Secondly, it is explored whether depression mediates the relationship between parental burnout and work outcomes. Findings suggest parental burnout may have limited impacts upon work outcomes, providing the impetus for a new direction of research to better understand whether or how burnout in one domain of life can influence the outcomes in other life domains.

**Supplementary Information:**

The online version contains supplementary material available at 10.1007/s12144-021-02687-3.

## Introduction

**Fig. 1:**
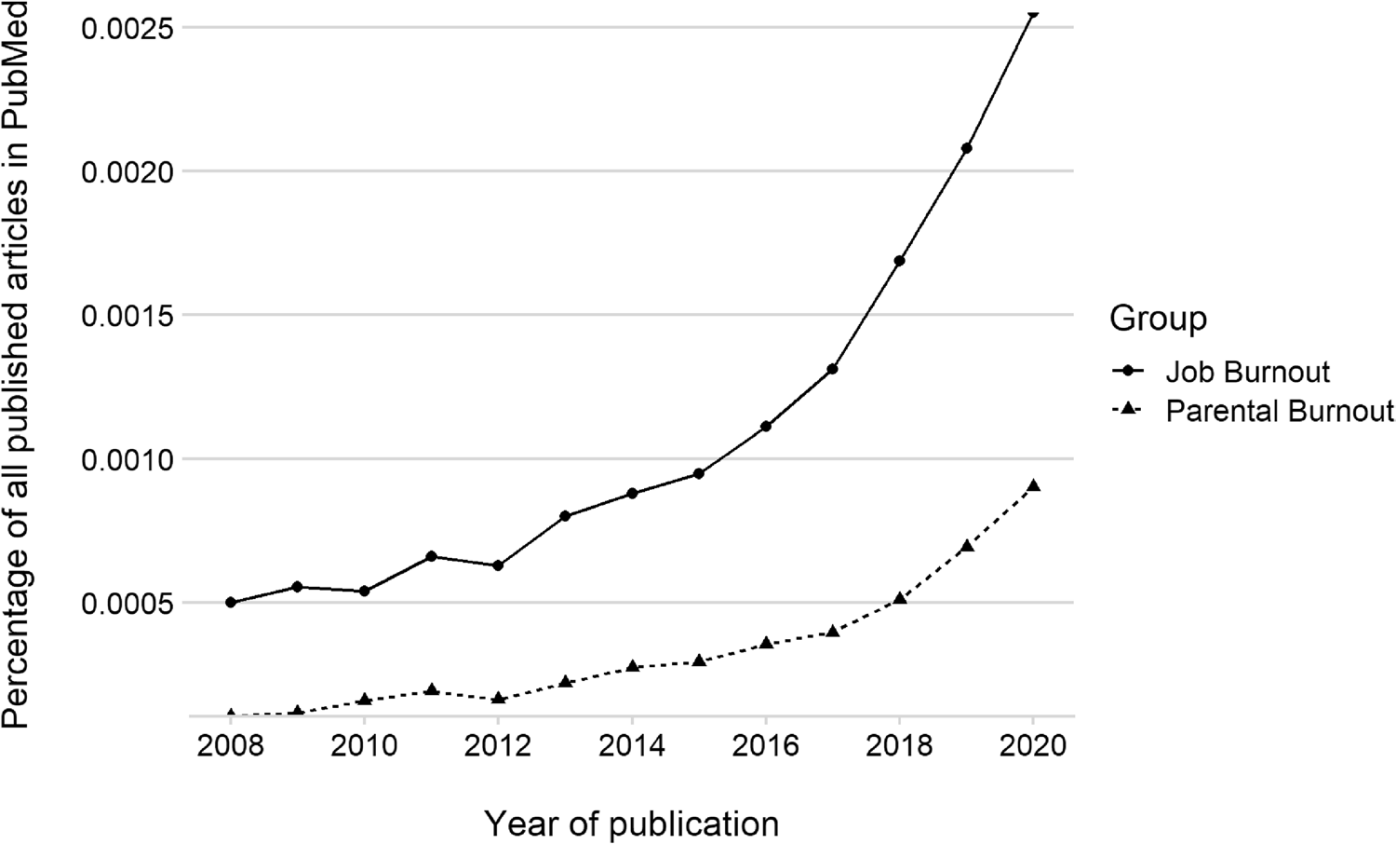
Growing interest in the fields of burnout

Burnout is typically considered a context-specific disorder (Bakker et al., 2000; Schaufeli et al., 2009), representing exhaustion, depersonalization and reduced personal accomplishment in the field in which it is experienced. Studies of burnout were originally focused upon the occupational context where it is still the most popularly researched domain (see Fig. 1). However, it has been increasingly considered in sporting (Raedeke & Smith, 2001), and parenting (Mikolajczak et al., 2019) contexts, among others.

The current work will focus upon the two most commonly experienced forms: job burnout and parental burnout. These different burnout types are typically represented with similar factor structures (exhaustion, depersonalization and [lack of] accomplishment) and some similar consequences have been reported e.g., substance abuse and poor sleep quality (Mikolajczak, Brianda, et al., 2018). However, the different burnout types form distinct factors and have been associated with a wide range of domain-congruent outcomes (Mikolajczak et al., 2020). For example, job burnout has been associated with a smorgasbord of negative personal and organizational consequences including decreased organizational commitment and performance, and increased absenteeism and turnover intentions (e.g., Lee & Ashforth, 1996; Swider & Zimmerman, 2010). Similarly, parental burnout has been associated with a range of parental behaviors and outcomes including family escape ideation, child neglect, and violent parenting (Mikolajczak, Raes, et al., 2018). However, there still remains a lack of clear evidence on whether burnout can result in domain-incongruent outcomes. The current study therefore aims to promote a more substantive evidence base by exploring whether parental burnout can influence job satisfaction, turnover intentions and counterproductive work behavior, and whether mental health captured through depression symptomatology could represent a key mechanism for such domain-incongruent outcomes.

### Theoretical and Empirical Foundations of Domain-Incongruent Effects of Burnout

According to Boundary theories, individuals tend to, although to differing extents, segment different identities and components of their lives (e.g., Clark, 2000). As such, we might expect that burnout experienced in one domain of life will be unlikely to feed substantively into another. For example, individuals experiencing high levels of parental burnout will perform at work to a mostly similar efficacy of those without parental burnout. Despite the growing body of evidence exploring domain-congruent outcomes, insufficient empirical attention has been received to draw robust conclusions on the extent to which burnout in one domain can directly impact outcomes in another.

Claims for direct associations between burnout and domain-incongruent outcomes are infrequent (e.g., Bianchi et al., 2014) and represent a limited body of evidence. In a recent exception to the majority of studies which report only domain-congruent outcomes, Mikolajczak et al. (2020) found little evidence of domain-incongruent outcomes for the different burnout types when studied alongside each other. Other studies have also reported similar small or non-significant effects concurrent with Boundary theories. For example, there is a non-significant relationship between parental burnout and career ambition (*r* (169) = -0.04; Meeussen & Van Laar, 2018). However, there are some sources of evidence to suggest that some direct domain-incongruent effects may be observable and impactful. For instance, high parental burnout has been associated with slightly *better* workplace performance (*β* (521) = 0.10), potentially due to work representing a safer or happier and thus more motivating environment (Mikolajczak et al., 2020). Our ability to draw conclusions surrounding the domain specificity of burnout is currently highly limited by the dearth of robust evidence exploring domain-incongruent outcomes. While far from conclusive, such mixed findings warrant exploration of small domain-incongruent effects further, particularly under a theoretical lens which can explain domain-incongruent and domain-general effects of the different burnout types.

#### Job Demands-Resource Theory

A popular framework for studying job burnout is the Job Demands-Resource Theory, which proposes that burnout results from inadequate resources to deal with demands (Bakker & Demerouti, 2007, 2017; Demerouti et al., 2001). While originally conceived in the workplace context, this model has been concurrent with many works from the parenting domain where burnout has been represented through an imbalance between demands and resources (Mikolajczak & Roskam, 2018). This model is capable of accounting for domain-incongruent effects by stating that burnout can drain important domain-general resources needed to cope with demands from all aspects of life. For example, Sandström et al. (2005) compared 67 patients with chronic burnout syndrome and 15 control participants to conclude that burnout may compromise cognitive abilities.

In both work and family domains, there are many personal resources (e.g., mental health, social support) which are seen as important to counterbalance the effects of excessive demands (e.g. Lin et al., 2021). Burnout from any life domain has the potential to compromise the resources available to an individual for domain-incongruent stress management (Mikolajczak & Roskam, 2018). The Job Demands-Resource Model may therefore be a useful framework to study such domain-incongruent outcomes through an indirect mediation effect which can acknowledge the impact of burnout on general resources. For example, while parental burnout may not directly compromise job performance, it may detract from the resources available to function optimally at work. Here, personal resources reflect the mechanism by which the different burnout types can impact domain-incongruent outcomes.

#### Depression and Burnout

Symptoms of depression like problematic sleep have been some of the most common outcomes representing resource loss from burnout in both job and parenting domains (e.g., Schonfeld & Bianchi, 2016). For example, Hakanen and Shaufeli (2012) found job burnout to predict future depression, with similar cross-sectional support for such relationships from parental burnout (Kawamoto et al., 2018; Van Bakel et al., 2018). Context-free and pervasive (Maslach et al., 2001), depression symptomatology could represent one key mechanism by which burnout impacts domain-incongruent outcomes. It might be expected that parental burnout may increase depressive symptomatology (Mikolajczak et al., 2020), which would in turn impact a wide range of outcomes including those within and beyond the occupational context. This mediation pathway for domain-incongruent outcomes (burnout—> lower resources—> poorer outcomes), is consistent with the Job Demands-Resource Theory but has yet to receive substantive empirical examination. Studying depression symptomatology in this context therefore represents a particularly promising line of inquiry.

### Aims and Hypotheses

**Fig. 2:**
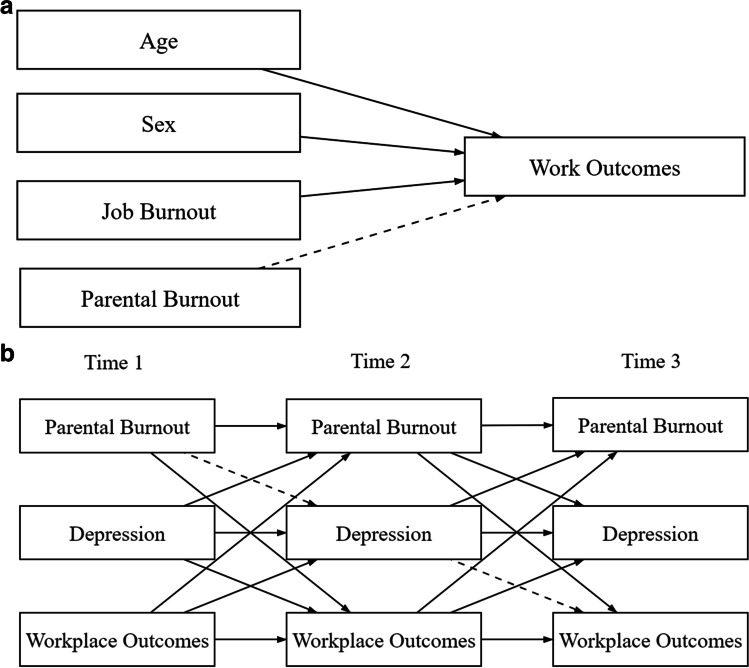
Hypothesized models for hypotheses (relationships of interest in dotted lines)

The current study explores the potential consequences of parental burnout for work outcomes in a longitudinal study of parents. We attempted to address two key discussions raised by the extant literature—whether parental burnout can directly impact outcomes in the occupational domain over and above variance explained by job burnout, and whether this may occur indirectly through depleted resources, e.g., depression symptomatology (Fig. 2).

- H1: After controlling for sex, age, and job burnout, parental burnout will explain additional variance in: a) job satisfaction, b) turnover intentions, and c) counterproductive work behavior.
- H2: Depression symptomatology will mediate the relationship between parental burnout and: a) job satisfaction, b) turnover intentions, and c) counterproductive work behavior.

## Materials and Method

### Design

To determine whether parental burnout can directly or indirectly impact work outcomes, a longitudinal self-report questionnaire design was adopted. The data analyzed represent a portion of a larger data set published elsewhere (Roskam & Mikolajczak, 2020). The data used for the current analyses, alongside the code and materials adopted, can be found at osf.io/krsua. The original study received the approval of the Institutional Review Board at the Université Catholique de Louvain.

### Participants

To participate, individuals had to be over 18, employed, and have at least one child. Participants were recruited through Prolific.ac (Palan & Schitter, 2018) and were required to complete a battery of online questionnaires three times, each four-months apart (November 2017, March 2018 and July 2018). Participants were paid £3 (GBP) for completion at each time-point and responses between time points were linked using Prolific ID numbers. Of the 874 participants recruited in total, 499 participants correctly answered questions confirming they were paying attention to the study instructions and completed the first and last battery of questionnaires. 421 completed all three. 281 of these participants were female, the mean age was 39.45 (*SD* = 7.63) and the sample represented parents with 1 to 6 children (*M* = 1.86, *SD* = 0.88).

### Materials

All participants completed the following battery of questionnaires, in addition to demographic questions, at all three time points. A copy of all available measures can be found in the supplementary materials (osf.io/98rmh). The mean, standard deviation and Alpha and Omega internal reliability estimates for each measure are noted in Table 1.

**Table 1:** Descriptive statistics and correlation matrix

	M	SD	a	o	1	2	3	4	5	6	7	8	9	10	11	12	13	14	15	16	17
1 – T1 Parental Burnout	29.20	22.67	.96	.96																	
2 – T2 Parental Burnout	26.97	20.46	.95	.96	.79																
3 – T3 Parental Burnout	27.31	20.91	.95	.96	.75	.81															
4 – T1 Job Burnout	36.87	18.00	.91	.90	.46	.44	.37														
5 – T2 Job Burnout	36.38	17.94	.91	.91	.41	.45	.39	.82													
6 – T3 Job Burnout	36.30	17.97	.91	.91	.43	.49	.44	.78	.83												
7 – T1 Depression	14.19	5.42	.94	.94	.53	.50	.44	.55	.51	.53											
8 – T2 Depression	13.94	5.39	.94	.94	.49	.50	.46	.51	.58	.55	.81										
9 – T3 Depression	13.80	5.28	.94	.94	.47	.52	.47	.47	.52	.57	.77	.81									
10 – T1 CWB	1.70	.91	.89	.89	.36	.37	.32	.44	.41	.41	.35	.32	.27								
11 – T2 CWB	1.63	.75	.84	.84	.31	.40	.30	.45	.47	.42	.25	.33	.25	.72							
12 – T3 CWB	1.67	.88	.89	.89	.28	.36	.31	.40	.44	.44	.30	.36	.34	.73	.74						
13 – T1 Turnover Intention	2.81	2.14	.96	.96	.29	.26	.21	.66	.59	.57	.40	.38	.34	.40	.41	.36					
14 – T2 Turnover Intention	2.74	2.05	.96	.96	.27	.29	.24	.60	.65	.59	.33	.40	.33	.38	.50	.43	.76				
15 – T3 Turnover Intention	2.80	2.13	.96	.96	.28	.32	.27	.57	.61	.68	.39	.43	.42	.34	.44	.40	.70	.80			
16 – T1 Job Satisfaction	4.93	1.64	.92	.92	-.27	-.28	-.21	-.72	-.67	-.63	-.38	-.39	-.34	-.28	-.31	-.27	-.72	-.64	-.60		
17 – T2 Job Satisfaction	4.96	1.64	.93	.93	-.24	-.26	-.24	-.64	-.74	-.67	-.31	-.39	-.35	-.32	-.37	-.35	-.58	-.72	-.64	.77	
18 – T3 Job Satisfaction	4.93	1.64	.94	.94	-.28	-.34	-.26	-.63	-.71	-.75	-.37	-.41	-.40	-.30	-.39	-.36	-.58	-.71	-.75	.75	.83

*Note: T1* = *Time 1; T2* = *Time 2; T3* = *Time 3; CWB* = *Counterproductive Work Behavior; N* = *421/499; a* = *Cronbach’s Alpha; o* = *Omega; All correlations p* < *.01*

*Parental burnout* was measured through the 22-item Parental Burnout Inventory (Roskam et al., 2017). This scale was developed to represent emotional exhaustion (8 items), emotional distancing (8 items), and loss of parental accomplishment and efficacy (6 items). Every item was responded to on a seven-point Likert-type scale ranging from never (0) to every day (6). A global parental burnout score was computed by summing the item scores after reversing those of the personal accomplishment factor, so that higher scores indicate greater parental burnout.

*Job burnout* was measured using the 16-item Maslach Burnout Inventory-General Survey (Schaufeli et al., 1996). The scale represents emotional exhaustion (5 items), cynicism (5 items) and professional efficacy (6 items). Again, every item was responded to on a seven-point Likert-type scale ranging from never (0) to every day (6). A global job burnout score was computed by summing the item scores after reversing those of the personal accomplishment factor, so that higher scores indicate greater job burnout.

*Depression symptomatology* was assessed through the PHQ-8 (Kroenke et al., 2009). Excluding suicidal thoughts, these eight items captured the experience of the DSM-5 depression criteria over the last month on a four-point Likert-type scale ranging from not at all (1) to nearly every day (4). The global score was obtained by summing all items.

*Job satisfaction* was captured through three items of the Job Satisfaction Index (Quinn & Shepard, 1974). Each item was responded to on a seven-point Likert scale ranging from strongly agree (1) to strongly disagree (7). The global score was obtained by averaging the item scores.

*Turnover intention* was measured through three items previously adopted by Lichtenstein et al. (2004). Each item was responded to through an eight-point Likert-type scale ranging from never (1) to a few times a day (8). The global score was obtained by averaging the item scores.

*Counterproductive work behaviors* were captured through seven items of the Counterproductive Work Behavior Checklist (Spector et al., 2006). Such items represent both organizational and person-directed behavior, including withdrawal, production deviance and abuse. Each item was responded to using an eight-point Likert-type scale ranging from never (1) to a few times a day (8). The global score was obtained by averaging the item scores.

### Data Analysis Plan

Following examination of required assumptions, relationships between all variables were captured through Spearman’s correlation coefficients (see Table 1). Multiple regression analyses explored the incremental predictive validity of parental burnout over job burnout for the prediction of work outcomes. Following this, longitudinal mediation explored the extent to which depression symptomatology mediates the relationship between parental burnout and work outcomes.

## Results

### Assumptions

The data set was first checked to explore the required assumptions. Univariate outliers were explored through z-scores. There were a few extreme outliers for counterproductive work behaviors and parental burnout, and indeed some are to be expected in large data sets (Selst & Jolicoeur, 1994). Univariate normality was assessed through skewness and kurtosis. Many of the latent variables presented high skewness/kurtosis scores (Wright & Herrington, 2011), indeed deviation from normal distribution was expected for many outcomes, for example, positive skew for parental burnout (Van Bakel et al., 2018). Multivariate outliers were examined through Mahalanobis Distance, where 30 participants scores were below the acceptable threshold (0.001). Finally, multicollinearity was calculated and no meaningful multicollinearity (VIF > 10) was identified. The minor violations of assumptions were considered acceptable, and thus no data was removed to preserve important characteristics of the data set, and to make use of all possible information (von Hippel, 2013).

### Regression Analyses

To explore whether parental burnout provides incremental predictive validity over job burnout for the prediction of work outcomes, three regressions were conducted using R (R Core Team, 2019). Full syntax and outputs can be found at osf.io/krsua. Predicting the work outcomes (Time 3), the first stage included only demographic information, the second included job burnout (Time 1), and the third included parental burnout (Time 1). Beta values presented are standardised and all values are presented alongside 95% confidence intervals. Findings from the three models are presented in Table 2, and suggest parental burnout provides little, if any, meaningful contribution to the prediction of work outcomes above that of job burnout. No additional variance in job satisfaction or turnover intentions was predicted by parental burnout, thereby providing no supportive evidence for H1a and H1b. Very modest support was provided for H1c where parental burnout predicted an additional 1% of variance in counterproductive work behaviors.

**Table 2:** Predicting work outcomes from job burnout and parental burnout

Outcome	Age	Sex	Job Burnout	Parental Burnout	R^2^	ΔR^2^
Job Satisfaction	.08 [-.01, 0.2]	-.09 [-.17, .00]			.01 [.00, .04]	
	-.02 [-.09, .05]	-.04 [-.11, .03]	-.63** [-.70, -.56]		.40** [.34, .46]	.39** [.32, .46]
	-.02 [-.09, .05]	-.04 [-.011, .03]	-.64** [-.72, -.56]	.02 [-.06, .09]	.40** [.34, .46]	.00 [-.00, .00]
Turnover Intention	-.12** [-.21, -.03]	.07 [-.02, .16]			.02 [.00, .05]	
	-.03 [-.11, .04]	.02 [-.05, .10]	.56** [.49, .64]		.33** [.26, .39]	.31** [.24, .38]
	-.03 [-.10, .04]	.02 [-.05, .10]	.55** [.47, .64]	.02 [-.06, .11]	.33** [.26, .38]	.00 [-.00, .00]
Counterproductive Work Behavior	-.19** [-.27, -.10]	.21** [.12, .29]			.08** [.04, .13]	
	-.13** [-.21, -.05]	.18** [.10, .26]	.36** [.28, .44]		.21** [.15, .27]	.13** [.07, .18]
	-.13** [-.21, -.05]	.18** [.10, .26]	.31** [.22, .40]	.12* [.03, .20]	.22** [.16, .28]	.01* [-.01, .03]

*Note: N* = *499;* * = p < .05; ** = p < .01

To negate the interpretation that parental burnout does not provide incremental predictive value because it represents the same psychological state as job burnout, the same analyses were conducted introducing parental burnout in the second stage and job burnout in the third. The findings are presented in Table 3 and suggest that job burnout predicts incremental variance in work outcomes over parental burnout. The findings therefore provide evidence for their distinctiveness by demonstrating how domain-congruent burnout provides the strongest predictions of outcomes.

**Table 3:** Predicting Work Outcomes from Parental Burnout and Job Burnout

Outcome	Age	Sex	Parental Burnout	Job Burnout	R^2^	ΔR^2^
Job Satisfaction	.08 [-.01, 0.2]	-.09 [-.02, .00]			.01 [.00, .04]	
	.05 [-.03, .14]	-.07 [-.16, .01]	-.27** [-.35, -.18]		.09** [.04, .13]	.07** [.03, .11]
	-.02 [-.09, .05]	-.04 [-.01, .03]	.02 [-.06, .09]	-.64** [-.72, -.56]	.40** [.34, .46]	.32** [.25, .38]
Turnover Intention	-.12** [-.21, -.03]	.07 [-.02, .16]			.02 [.00, .05]	
	-.09 [-.18, -.01]	.06 [-.03, .14]	.27** [.19, .36]		.09** [.05, .14]	.07** [.03, .12]
	-.03 [-.10, .04]	.02 [-.05, .10]	.02 [-.06, .11]	.55** [.47, .64]	.33** [.26, .38]	.24** [.17, .30]
Counterproductive Work Behavior	-.19** [-.27, -.10]	.21** [.12, .29]			.08** [.04, .13]	
	-.16** [-.25, -.08]	.20** [.11, .28]	.25** [.17, .33]		.15** [.09, .20]	.06** [.02, .10]
	-.13** [-.21, -.05]	.18** [.10, .26]	.12* [.03, .20]	.31** [.22, .40]	.22** [.16, .28]	.07** [.03, .12]

*Note: N* = *499;* * = p < .05; ** = p < .01

### Complete Longitudinal Mediation Analyses

Analysis of the longitudinal mediation was conducted through Mplus 6 (Muthén & Muthén, 2010) following recommendations and syntax by Jose (2016). The complete longitudinal mediation analysis model was adopted, whereby all variables were represented in the models at all time points, and all possible mediation pathways were tested. Only the main mediation pathways of interest are reported in this article. However, all analysis syntax and full outputs can be found at osf.io/krsua. Models were evaluated with respect to the degree to which they approximate the data. The following goodness of fit indices and cut-offs are presented. Fit to the data was considered adequate with values of ≤ 0.08 for the RMSEA (Browne & Cudeck, 1992) and SRMR (Hu & Bentler, 1999), and ≥ 0.90 for the CFI and TLI, (Bentler & Bonett, 1980) with values above 0.95 preferred (Hu & Bentler, 1999).

#### Model 1: Job Satisfaction

Analyses explored the longitudinal mediation of depression in the relationship between parental burnout and job satisfaction. Model fit was acceptable (RMSEA = 0.00; CFI = 1.00; TLI = 1.00; SRMR = 0.00). Counter to H2a, parental burnout at Time 1 was not significantly associated with depression at Time 2 (*β* = 0.05, *p* = 0.21) and depression was not significantly associated with job satisfaction at Time 3 (*β* = -0.03, *p* = 0.44). No significant indirect relationship was reported (*β* = -0.00, p = 0.56).

#### Model 2: Turnover Intentions

Analyses explored the proposed mediation model for turnover intentions. Model fit was acceptable (RMSEA = 0.00; CFI = 1.00; TLI = 1.01; SRMR = 0.00). Counter to H2b, parental burnout at Time 1 was not significantly associated with depression at Time 2 (*β* = 0.05, *p* = 0.22) but depression at Time 2 was significantly associated with turnover intentions at Time 3 (*β* = 0.09, *p* = 0.01). No significant indirect relationship was found (*β* = 0.00, *p* = 0.29).

#### Model 3: Counterproductive Work Behavior

Analyses explored the same model for counterproductive work behaviors. Model fit was acceptable (RMSEA = 0.04; CFI = 1.00; TLI = 0.99; SRMR = 0.01). Counter to H2c, parental burnout at Time 1 was not significantly associated with depression at Time 2 (*β* = 0.04, *p* = 0.27). However, depression at Time 2 was significantly associated with counterproductive work behaviors at Time 3 (*β* = 0.11, *p* = 0.01). As such, no mediation was found (*β* = 0.01, *p* = 0.31).

## Discussion

### Main Findings

Analyzing a longitudinal data set of nearly 500 participants, parental burnout provided no incremental validity above job burnout for the prediction of job satisfaction and turnover intentions, but explained an additional 1% of variance in counterproductive work behavior. These results provide little support for the direct relationship between parental burnout and work outcomes in Hypotheses 1a-c. Furthermore, depression symptomatology did not mediate the relationship between parental burnout and work outcomes. Hypotheses 2a-c regarding an indirect relationship therefore also received no support. As a whole, results suggest that parental burnout has little-to-no direct impact upon work outcomes. If such a relationship is indirect it is unlikely to occur through a depression pathway.

### Theoretical Implications

These findings could be interpreted as inconsistent with the Job Demands-Resource Model (Bakker & Demerouti, 2007, 2017; Demerouti et al., 2001) whereby we might have expected domain-incongruent effects of parental burnout via the suppression or weakening of personal resources needed to deal with stress. The lack of domain-incongruent outcomes supports the general view that burnout is context-specific (Bakker et al., 2000; Schaufeli et al., 2009) and is concurrent with Boundary theories in proposing that different domains of life are generally separated (Clark, 2000).

An alternative interpretation consistent with the Job Demands-Resource Model might be that this weakening of personal resources due to parental burnout is compensated by the change of a resource in the job demands/resource balance: namely, a renewed appreciation of one’s job. The drain of personal resources due to burnout in one domain could be compensated by the renewed engagement or satisfaction in a different domain. Such an interpretation would be incompatible with depression as a central resource in this process based upon current findings; yet the premise is concurrent with the small increase in job satisfaction associated with parental burnout reported by Mikolajczak et al. (2020).

Only one set of personal resources, captured through depression symptomatology, was represented in the current study. Contrary to the extant literature which typically reports strong relationships and describes the constructs as inter-related (e.g., Bianchi et al., 2021; Maslach & Leiter, 2016; Schonfeld & Bianchi, 2016), parental burnout was not a significant predictor of depression. It is possible that the mainstream use of cross-sectional data has led to over-estimates of this relationship and that this longitudinal work has provided an important nuance: that parental burnout does not predict *future* depression. The relationship between parental burnout and depression is clearly complex, and thus further longitudinal research is needed to elucidate the exact causal paths as to how depression symptomatology, and other markers of personal resources, coalesce.

While providing little support for any of the hypotheses tested, the findings are interpretable in the context of the theoretical and empirical literature. The findings are also small in magnitude, in-line with the other studies exploring domain-incongruent consequences of burnout (e.g., Mikolajczak et al., 2020). As early work in this area, the current study yields a clear new direction for appreciating the intricacies and nuances associated with burnout and its consequences for domain-incongruent outcomes.

### Practical Implications

The current findings could be construed as a justification for ignoring the parental demands of employees. Such actions are ill-advised in the context of the significant body of evidence to suggest that organizational parental support can facilitate greater personal coping and subsequent occupational outcomes such as organizational commitment (Anderson et al., 2002; Grover & Crooker, 1995). As the legal organizational demands for organizations to support parents predominantly cover maternity/paternity leave (Moss & Deven, 2006), there may be scope for developments in this research field to inform more family-friendly working practices. Any early claims for the value of workplace interventions to support employees with parental burnout should be treated with extreme caution however. The current study, combined with early findings indicating increased job performance from individuals with high parental burnout (Mikolajczak et al., 2020), suggests that the organizational outcomes of parental burnout are likely to be complex, with small yet meaningful impacts likely. Thus, any practical conclusions drawn should be done so based upon evaluation of a much larger body of evidence than that which is currently available.

### Limitations

The current study has a substantive sample size, uses well-validated measures, and captures all constructs over a meaningful period of time. As such, the current study represents a relatively robust evaluation of the hypotheses explored. Nevertheless, the current study is one of the first longitudinal explorations of domain-incongruent impacts of burnout, and should be treated with a modest level of caution. In particular, the exclusive use of self-report questionnaires raises concerns surrounding common method bias and thus the possibility of distorted relationship estimates (Podsakoff et al., 2003). Self-report questionnaires were considered the most appropriate measurement method to capture each of the constructs discussed; reliability estimates and correlations have been detailed; the convergent/divergent validity of the measures has been established (Mikolajczak et al., 2019); and analyses focused upon relationships between, not within, time points. Best-practice recommendations to minimize any potential consequences of common method bias were therefore adopted (Conway & Lance, 2010).

The global COVID-19 pandemic has created an unprecedented turn to remote work, causing a subsequent strain upon work boundaries (Rudolph et al., 2021). This strain has been exacerbated by the additional caregiving demands placed upon parents during lockdown when many also provided home-schooling (Thorell et al., 2021). The effects of such a complex context have thus far provided mixed conclusions as to whether the dynamics between work and parenting have changed and, if so, for how long (Aguiar et al., 2021; Le Vigouroux et al., 2021). As the data reported in the current work were collected pre-pandemic, we therefore encourage great caution when attempting to apply our findings within context of the ongoing pandemic and encourage greater emphasis on longitudinal data collected during the pandemic to provide robust recommendations for supporting parents to help manage the many conflicting demands they continue to face.

### Future Research

There have been substantive debates and contradictions presented by the extant theoretical and empirical works on the relatedness of depression and burnout (e.g., Bianchi et al., 2015, 2021). This was similarly evidenced by the divergence in the nature of the relationship between parental burnout and depression between the current longitudinal work and prior cross-sectional research. As such, further longitudinal work will be particularly important for drawing robust explanatory and predictive integrative models in this field.

With respect to the impact of parental burnout upon work outcomes, future research should be encouraged to consider alternative resource-based mechanisms by which parental burnout can impact upon work outcomes. The Job Demands-Resources model represents an excellent theoretical framework to support future research to question whether parental burnout can exhaust personal resources such as self-efficacy and optimism, necessary for work outcomes (Bakker & Demerouti, 2007, 2017; Demerouti et al., 2001).

The current study highlights the relative absence of work exploring domain-incongruent outcomes of burnout, and in doing so raises an important new direction in burnout research. Acknowledging that the current findings reflect preliminary work and small effect sizes, further longitudinal work should attain appropriate sample sizes to draw robust estimates. The use of power analyses and similar data simulations which can inform sample size based upon the foci of interest are encouraged to evoke greater confidence in conclusions and minimize obfuscation of relationships. While often small in effect, exploring the impact of domain-incongruent outcomes of burnout represents an exciting opportunity to clarify the burnout process, and how it can be interrupted.3

## Supplementary Information

Below is the link to the electronic supplementary material.

Supplementary file1 (DOCX 15 kb)

## Data Availability

Data is openly available on the Open Science Framework: osf.io/krsua.
