# Supplementary material for: Burnout across boundaries: Can parental burnout directly or indirectly influence work outcomes?
Source: Curr Psychol. 2022 Jan 22:1–11. Online ahead of print. doi: 10.1007/s12144-021-02687-3 (PMC8783659; doi:10.1007/s12144-021-02687-3)
Supplement: Supplementary file 1 — Supplementary file1 (DOCX 15 kb) [file 12144_2021_2687_MOESM1_ESM.docx]

**Supplementary Materials**

All materials, data, analysis code, and outputs are openly available on the Open Science Framework: osf.io/krsua
